# Factors influencing the use of maternal healthcare services and childhood immunization in Swaziland

**DOI:** 10.1186/s12939-015-0162-2

**Published:** 2015-03-27

**Authors:** Mluleki Tsawe, Amos Moto, Thendo Netshivhera, Lesego Ralesego, Cassandra Nyathi, A Sathiya Susuman

**Affiliations:** Department of Statistics & Population Studies, University of the Western Cape, Cape Town, South Africa; Population Statistics Division, Statistics South Africa (StatsSA), Pretoria, South Africa; Population Unit, North West University, Mafikeng, South Africa; Department of Demography & Population Studies, University of the Witwatersrand, Johannesburg, South Africa; Department of Statistics & Population Studies, University of the Western Cape, Cape Town, South Africa

**Keywords:** Maternal healthcare, Antenatal care, Delivery care, Postnatal care, Child health, Immunization, Socio-demographic factors

## Abstract

**Background:**

Maternal and child healthcare services are very important for the health outcomes of the mother and that of the child and in ensuring that both maternal and child deaths are prevented. Studying these services is necessary in developing countries where infrastructure (which is meant to deal with these health services) is minimal or lacking. The objective of the study is to examine the factors that influence the use of maternal healthcare services and childhood immunization in Swaziland.

**Methods:**

Our study used secondary data from the Swaziland Demographic and Health Survey 2006–07. This is an explorative and descriptive study which used pre-selected variables to study factors influencing the use of maternal and child healthcare services in Swaziland. We ran three different types of analyses: univariate, bivariate and multivariate. For the multivariate analysis, a logistic regression was run to investigate the relationship between the dependent and independent variables.

**Findings:**

The study findings showed a high use rate of antenatal care (97.3%) and delivery care (74.0%) and a low rate of postnatal care use (20.5%). The uptake of childhood immunization is also high in the country, averaging more than 80.0%. Certain factors which were found to be influencing the use of maternal healthcare and childhood immunization include: woman’s age, parity, media exposure, maternal education, wealth quintile, and residence. The findings also revealed that these factors affect the use of maternal and child health services differently.

**Conclusion:**

It is important to study factors related to maternal and child health uptake to inform relevant stakeholders about possible areas of improvement. Programs to educate families about the importance of maternal and child healthcare services should be implemented. In addition, interventions should focus on: (a) age differentials in use of maternal and child health services, (b) women with higher parities, (c) women in rural areas, and (d) women from the poor quintile. We recommend that possible future studies could use the qualitative approach to study issues associated with the low use of postnatal services.

## Background

The Kingdom of Swaziland (hereon referred to as ‘Swaziland’) is a small country in Southern Africa neighbouring Mozambique and South Africa. Swaziland is a monarchy, with an estimated population size of 1 231 000 inhabitants as estimated in 2010 [1]. This is a relatively small country, compared to various other countries in sub-Saharan Africa, with the exception of Lesotho. In 2008, the life expectancy at birth was estimated at 48 years [2]. Furthermore, the total fertility rate is estimated at 3.8 births per woman, where rural women contribute more (4.2) births per woman as compared to urban women (at 3.0 births per woman) [3]. According to the 2010 estimates, the maternal mortality ratio was 320 maternal deaths per 100 000 live births; this is a considerable reduction (with a percentage decrease of 23.8%) from the maternal mortality ratio estimated five years earlier at 420 maternal deaths per 100 000 live births [4]. Nonetheless, this maternal mortality ratio is still relatively high, and has some serious implications for Swaziland’s prospects of achieving the fifth Millennium Development Goal (MDG). The fifth MDG advocates for improvements in maternal health and the reduction of maternal mortality.

In 2012, Swaziland’s under-five mortality rate (U5MR) was estimated at 80 deaths per 1000 live births [5]. Immunization, as part of the fourth MDG, is central to the world agenda to improve child health worldwide. The mandate of MDG4 is for all countries to reduce their U5MR by two-thirds by the year 2015 [6]. Complete immunization of children is necessary to combat preventable diseases and reduction of infant as well as child mortality [3]. Several developing countries have managed to work well and increase the uptake of maternal and child health services, but there is still more that needs to be done to lessen inequalities in terms of universal coverage and use among people of different geographical locations (rural and urban), socio-economic statuses, as well as other significant demographic and socio-economic factors [6-8]. Previous studies have noted that several socio-economic and demographic factors affect the use of maternal and child healthcare services (more on this will be discussed further in the study).

### Maternal healthcare

There are various socio-demographic factors that influence and cause a difference in the utilization of maternal health services. The following factors have been widely documented in the literature and are known to affect the use of maternal health services across the world: maternal education, woman’s age, employment and income, socio-economic status, residence (i.e. rural/urban), parity, distance to health facilities, and exposure to the media [9-13]. Regarding parity, it is generally argued that those who have more than three living children tend to believe that they are more experienced to handle their maternal and reproductive health issues; as such, they utilize maternal health services less frequently as compared to those who have had less than three children [13,14]. This list is only but a few of the influences of the uptake of maternal health services – some of these influences are not listed here as the list would be endless.

### Childhood immunization

The sub-Saharan Africa region is among the regions with the highest under-five mortality rate – it is said to contribute over sixteen times the average of developed regions [6]. Swaziland’s U5MR showed a percentage increase of 70.4% between 1990 and 2000, from 71 deaths per 1000 live births to 121 deaths per 1000 live births, respectively; but between the year 2000 and 2012, there has been a percentage decrease of 33.9% in U5MR – which shows that the country is progressing towards reaching the target of 24 child deaths per 1000 live births by 2015 [5]. Certain factors have been found to influence the complete use of immunization. These factors can be barriers to the prospect of achieving MDG4 in developing countries. Authors argue that maternal education, exposure to the media, maternal age, decision-making autonomy and household economic status influence the uptake of childhood immunization [15,16]. A study by [17] found that children whose mothers have the freedom to make decisions were likely to be fully immunized. With regard to maternal education, secondary education has been found to increase the use of immunization [15]. It has also been found that utilization of immunization is lower among children whose mothers have lower (i.e. primary) or no education in Nigeria [17].

### Urban–rural differences in maternal and child healthcare use

According to [3] there are more women of reproductive age in rural areas (73.3%) as compared to urban areas (26.7%). In light of this, it is important to understand the consequences of these differentials. Most countries, even developing countries, have bigger urban populations than rural populations, due to rural-to-urban migration. In relation to urban–rural differentials in maternal healthcare use, it is generally believed that women who reside in urban areas are more likely to make use of the available maternal health services as compared to those who reside in rural areas [12,18,19]. Similarly, a study in Nigeria found that a relationship exists between place of residence and the probability of children receiving complete immunization – where only 9% of children in rural areas received full immunization while the same can be said for 34% of children in urban areas [20]. These results are usually associated with countries (or communities) that have more women in urban areas as compared to those in rural areas. Moreover, in developing countries the delivery of healthcare services tends to be unequally spread between rural and urban areas, where the most state-of-the-art resources often favour those in urban areas as compared to those in rural areas [21]. For instance, the socio-economic status of women in rural areas tends to be lower than that of women in urban areas. The lack or minimal use of maternal and child healthcare services by rural women is often attributed to issues of accessibility and affordability – whereby women in these areas have to travel long distances before they reach the nearest heath facility [22-24].

### Statement of the problem

Swaziland is among the lower-middle-income economies in Africa with a large proportion of its population still living in poverty. Disparities between those who *have* and those who *do not have* are not unexpected in the country. Moreover, the maternal mortality ratio (MMR) is high for a country with a total population of just over a million inhabitants. It is essential to examine the factors that may be contributing to this high MMR (whether related to maternal health or child health). Though the country has a high MMR, the use of institutional deliveries is relatively high, with a reported rate of less than three quarters (74%) [3]. This rate suggests that more than a quarter of (26%) births in Swaziland are non-institutional deliveries – which is a very high number. International organizations are advocating for complete use of institutional deliveries and most countries are struggling to meet this quota. The data shows that the country is doing well with its provision of maternal healthcare services, except for postnatal care – with three quarters of the women not making use of postnatal care services [3]. The importance of maternal healthcare services and childhood immunization cannot be stressed enough. These services are very important for the health and health outcomes of the mother and that of the child and in ensuring that both maternal and child deaths are prevented. Moreover, studying these services is necessary in developing countries where infrastructure (which deals with these health services) is minimal or lacking. The authors decided to study Swaziland because of the scarcity of studies relating to maternal and child healthcare services in the country. Based on our knowledge – there is no other study that has holistically focused on both of these important aspects (maternal and child health) in Swaziland.

### Objectives

The main objective of the study is to examine the factors that influence the use of maternal healthcare services and childhood immunization in Swaziland.

## Data & methods

### Data

We used secondary data (weighted) drawn from the 2006–07 Swaziland Demographic and Health Survey – which was the first large-scale survey to be conducted in the country [3]. The survey collected demographic and social information from a nationally representative sample of 4,987 women of reproductive age (15–49), with a response rate of 94.1% [3]. Most developing countries, especially in Africa, struggle with the acquisition of data related to maternal and child health. The demographic and Health Surveys are a source of reliable health data pertaining to maternal, child health, and general health of households surveyed. This is an explorative and descriptive study which uses pre-selected variables to study factors influencing the use of maternal and child healthcare services in Swaziland. In this study, the data are analysed using SPSS (Statistical Package for the Social Sciences) version 16.0.

### Univariate analysis

The univariate analysis (Table 1) comprises of a frequency distribution of five selected variables which are central to this study. This distribution profiles the categories (through percentages) within each variable.

**Table 1:** **Distribution of selected background characteristics based on women of reproductive age (15–49) in Swaziland**

**Variables**	**n**	**%**
*Woman’s age*		
15-19	268	2.4
20-24	1131	9.9
25-29	1559	13.7
30-34	1949	17.1
35-39	2097	18.4
40-44	2273	20.0
45-49	2106	18.5
*Antenatal care*		
No visits	55	2.6
All visits	2016	97.4
*Delivery care*		
Non-institutional	730	25.8
Institutional	2094	74.2
*Postnatal care*		
No	405	79.4
Yes	105	20.6
*Immunization*		
No	68	11.7
Yes	516	88.3

Source: Authors calculations from the Swaziland Demographic and Health Survey 2006–07.

#### Operational definition(s)

For purposes of this study, the outcome variables are defined as follows:

These services are those provided by health care professionals within a health-facility setting.

- Antenatal care (numbers of visits to the health care center during pregnancy);
- Delivery care (the place where the women gave birth)
- Postnatal care (baby postnatal care within two months after delivery)
- Immunization (taken as those children who have had a vaccination).

### Bivariate analysis

#### Selected variables

For the bivariate analysis, the variables were selected based on literature from various other studies which studied factors associated with maternal and child healthcare use. The main focus of this study was on four variables: *antenatal care* (antenatal visits for pregnancy); *delivery care* (place of delivery); *postnatal care* (baby postnatal care within two months); *immunization* (ever had vaccination). These variables also serve as the dependent variables for the multivariate analysis. In addition, the focus of the bivariate analysis was on the Chi-square (χ^2^) test and p-values which the authors employed to present the study findings. The χ^2^ test was used to test whether an association exists between the four main variables of this study and the independent variables. In addition to the variables mentioned above, there are ten pre-selected variables which are also used as independent variables for the multivariate analysis. These are: woman’s age (in five-year groups); parity; exposure to newspapers (or magazine); exposure to the radio; exposure to television; maternal education; wealth quintile; literacy; and residence.

#### Multivariate analysis

A logistic regression model is employed to study the relationship between the independent variables (listed above) and four dependent variables. All the dependent variables selected for the multivariate analysis are dichotomous (i.e. have two categories). The independent variables (mentioned above, see bivariate analysis), are categorical, except for *literacy*, and *residence*. The dependent variables are: antenatal care (0 = no visits and 1 = one or more visits); delivery care (0 = non-institutional delivery, 1 = institutional delivery); postnatal care (0 = No, 1 = Yes); and immunization (0 = No, 1 = Yes). Please note, some of the variables were excluded in the analysis because they had extremely large standard errors which affected the odds ratio. In addition, for the purpose of this study, non-institutional deliveries are defined as all births that occurred outside the conventional or official health facilities (i.e. those births which occurred at respondents home, NGO, etc.); and institutional deliveries are those births which occurred in health facilities (both public and private). The logistic regression model is used to examine the relationship (odds ratios) between the dependent variable and a set of pre-selected independent variables.

### Ethical considerations

This study is based on secondary analysis of data that is publicly available from the DHS Program’s website. Therefore, no ethical approval was required from our institutions.

## Results

This section presents results from the univariate (Table 1) bivariate (Tables 2 and 3) and multivariate (Table 4) analyses. The results (for both the bivariate and multivariate analysis) are based on the 95% confidence interval.

**Table 2:** **Distribution of women aged 15–49 by background variables related to maternal and child healthcare services (based on** ***p-values*** **and** ***Chi-square*** **)**

**Variables**	**Maternal health**						**Child health**	
	**Antenatal care**		**Delivery care**		**Postnatal care**		**Immunization**	
	**P-value**	**χ** ^**2**^	**P-value**	**χ** ^**2**^	**P-value**	**χ** ^**2**^	**P-value**	**χ** ^**2**^
*Woman’s age*	0.0**	34.9	0.0*	28.2	0.4	12.6	0.3	14.8
*Parity*	0.0*	20.2	0.0*	129.6	0.6	3.0	0.1	6.8
*Exposure to newspapers*	0.0**	21.2	0.0*	137.1	0.7	4.1	0.1	10.0
*Exposure to the radio*	0.0*	27.8	0.0*	25.3	0.4	6.3	0.0***	13.9
*Exposure to television*	0.0*	30.1	0.0*	134.6	0.1	12.3	0.2	8.5
*Maternal education*	0.0*	39.0	0.0*	227.7	0.1	10.7	0.5	5.3
*Wealth quintile*	0.0*	31.4	0.0*	302.7	0.0***	9.8	0.0*	24.6
*Literacy*	0.0***	8.2	0.0*	65.6	0.1	3.9	0.0***	5.9
*Residence*	0.1	6.0	0.0*	106.9	0.7	0.7	0.0**	10.2

Note: Significance level: * = P < 0.001; ** = P < 0.01; *** = P < 0.05.

- = Not applicable.

Source: Authors calculations from the Swaziland Demographic and Health Survey 2006–07.

**Table 3:** **Percentage of women (aged 15–49) by use of maternal and child healthcare services according to various background characteristics**

**Variables**	**Maternal health**						**Child health**	
	**Antenatal care**		**Delivery care**		**Postnatal care**		**Immunization**	
	**No visits**	**All visits**	**Non-inst.**	**Inst.**	**No**	**Yes**	**No**	**Yes**
***Woman’s age***								
15-19	2.6	97.4	22.6	77.4	80.0	20.0	11.3	88.7
20-24	2.2	97.9	23.3	76.7	84.6	15.4	11.6	88.4
25-29	3.0	97.0	24.2	75.8	76.5	23.5	11.2	88.8
30-34	3.3	96.7	26.5	73.5	77.5	22.5	13.6	86.4
35-39	1.3	98.8	32.6	67.4	71.4	28.6	6.4	93.6
40-44	4.3	95.7	34.5	65.5	88.1	11.9	5.3	94.7
45-49	3.8	96.1	50.0	50.0	85.7	14.3	10.0	90.0
***Parity***								
1-2 children	1.8	98.2	18.0	82.0	79.2	20.8	10.7	89.3
3-5 children	3.1	96.9	28.7	71.3	77.1	22.9	9.5	90.5
6 or more	4.9	95.1	44.5	55.5	83.2	16.8	18.1	81.9
***Exposure to newspapers***								
Not at all	3.9	96.2	35.9	64.1	80.7	19.3	14.5	85.5
Less than once a week	2.4	97.5	24.6	75.4	82.4	17.6	10.7	89.3
At least once a week	1.9	98.1	19.1	80.9	75.0	25.0	8.1	91.9
Almost every day	1.1	98.9	10.8	89.2	75.0	25.0	9.4	90.6
***Exposure to the radio***								
Not at all	4.3	95.7	32.9	67.1	80.4	19.6	14.3	85.7
Less than once a week	3.4	96.6	25.2	74.8	92.9	7.1	8.6	91.4
At least once a week	2.2	97.9	28.5	71.5	80.0	20.0	10.1	89.9
Almost every day	2.0	98.0	22.8	77.2	77.3	22.7	10.4	89.6
***Exposure to television***								
Not at all	2.8	97.3	32.7	67.3	81.9	18.1	13.1	86.9
Less than once a week	7.2	92.8	18.5	81.5	82.4	17.6	2.7	97.3
At least once a week	2.5	97.5	22.0	78.0	60.6	39.4	4.0	96.0
Almost every day	1.0	99.0	10.0	90.0	69.0	31.0	10.1	89.9
***Maternal education***								
No education	3.4	96.5	44.9	55.1	86.7	13.3	16.7	83.3
Primary	4.2	95.7	37.7	62.3	81.8	18.2	9.9	90.1
Secondary	1.4	98.6	16.6	83.4	72.2	27.8	10.9	89.1
Higher	2.8	97.2	4.0	96.0	75.0	25.0	13.0	87.0
***Wealth quintile***								
Poor	4.4	95.6	42.5	57.5	81.2	18.8	20.0	80.0
Middle	2.5	97.6	21.7	78.3	85.0	15.0	4.0	96.0
Rich	1.1	98.8	10.7	89.3	68.5	31.5	7.9	92.1
***Literacy***								
Illiterate	3.8	96.3	46.4	53.6	86.5	13.5	17.3	82.7
Literate	2.6	97.4	23.8	76.2	78.0	22.0	10.5	89.5
***Residence***								
Urban	1.8	98.2	11.5	88.5	77.0	23.0	6.2	93.8
Rural	3.0	97.0	31.1	68.9	79.9	20.1	14.3	85.7

Note: Non-inst. = Non-institutional; Inst. = Institutional.

- = Not applicable.

Source: Authors calculations from the Swaziland Demographic and Health Survey 2006–07.

**Table 4:** **Logistic regression (Odds Ratio = OR) of factors influencing the use of maternal and child healthcare services in Swaziland**

**Variables**	**Maternal health**			**Child health**
	**Antenatal care**	**Delivery care**	**Postnatal care**	**Immunization**
	**OR (95% C.I.)**	**OR (95% C.I.)**	**OR (95% C.I.)**	**OR (95% C.I.)**
***Woman’s age***				
15-19®	1	1	1	1
20-24	0.76 (0.1 - 3.9)	0.43 (0.1 - 2.4)	0.77 (0.1 - 5.5)	1.40 (0.5 - 4.0)
25-29	1.25 (0.2 - 10.0)	0.37 (0.1 - 2.1)	1.03 (0.1 - 7.9)	1.42 (0.4 - 4.6)
30-34	1.63 (0.1 - 27.9)	0.71 (0.1 - 4.3)	1.63 (0.2 - 13.7)	2.16 (0.5 - 9.2)
35-39	0.64 (0.0 - 10.3)	0.66 (0.1 - 4.2)	2.82 (0.3 - 24.2)	7.60*** (1.0 - 59.2)
40-44	0.76 (0.0 - 17.4)	0.89 (0.1 -6.4)	1.40 (0.1 - 15.5)	17.72*** (1.3 - 250.1)
45-49	0.44 (0.0 - 15.1)	0.27 (0.0 - 2.8)	2.23 (0.1 - 35.1)	2.79 (0.2 - 42.0)
***Parity***				
1-2 children®	1	1	1	1
3-5 children	4.90 (0.5 - 52.7)	0.53 (0.3 - 1.0)	1.08 (0.4 - 2.7)	1.23 (0.5 - 2.8)
6 or more	0.54 (0.0 - 6.3)	0.41*** (0.2 - 1.0)	0.44 (0.1 - 1.5)	0.20*** (0.0 - 0.9)
***Exposure to newspapers***				
Not at all®	1	1	1	1
Less than once a week	0.69 (0.2 - 3.0)	0.84 (0.4 - 1.7)	0.57 (0.2 - 1.6)	0.99 (0.4 - 2.6)
At least once a week	1.47 (0.3 - 7.8)	0.91 (0.5 - 1.7)	0.54 (0.2 - 1.3)	1.99 (0.9 - 4.5)
Almost every day	0.95 (0.2 - 5.7)	1.11 (0.5 - 2.6)	0.67 (0.2 - 2.2)	1.50 (0.6 - 3.8)
***Exposure to the radio***				
Not at all®	1	1	1	1
Less than once a week	0.45 (0.1 - 2.0)	0.98 (0.3 - 3.0)	1.18 (0.2 - 6.8)	1.97 (0.5 - 7.9)
At least once a week	0.94 (0.2 - 4.2)	1.19 (0.5 - 2.8)	0.86 (0.3 - 2.2)	1.66 (0.5 - 5.1)
Almost every day	1.99 (0.6 - 7.0)	1.12 (0.7 - 1.9)	0.73 (0.4 - 1.4)	1.07 (0.6 - 2.1)
***Exposure to television***				
Not at all®	-	1	1	1
Less than once a week	-	1.33 (0.5 - 3.6)	3.29 (0.6 - 18.9)	3.63 (0.5 - 29.2)
At least once a week	-	1.42 (0.6 - 3.3)	4.36** (1.5 -12.7)	1.64 (0.4 - 7.6)
Almost every day	-	1.93 (1.0 - 3.9)	0.87 (0.2 - 3.2)	0.76 (0.3 - 1.7)
***Maternal education***				
No education®	-	1	1	1
Primary	-	0.39 (0.1 - 1.1)	1.06 (0.3 - 4.0)	1.41 (0.3 - 6.3)
Secondary	-	0.75 (0.3 - 2.1)	1.58 (0.4 - 6.6)	0.50 (0.1 - 2.6)
Higher	-	1.03 (0.2 - 7.0)	1.07 (0.0 - 26.9)	0.23 (0.0 - 1.6)
***Wealth quintile***				
Poor®	1	1	1	1
Middle	1.68 (0.3 - 8.3)	1.96*** (1.1 - 3.6)	0.38 (0.1 - 1.1)	5.28* (1.7 - 16.3)
Rich	0.94 (0.3 - 3.5)	2.55* (1.3 - 4.9)	1.78 (0.7 - 4.6)	2.53*** (1.1 - 6.0)
***Literacy***				
Illiterate®	1	1	1	1
Literate	1.84 (0.5 - 7.3)	1.99 (0.7 - 5.5)	1.35 (0.4 - 5.1)	1.83 (0.4 - 7.4)
***Residence***				
Urban®	1	1	1	1
Rural	0.66 (0.2 - 2.8)	0.82 (0.4 - 1.5)	1.35 (0.4 - 4.1)	0.49 (0.2 - 1.0)

Note: ® = Reference category;

Significance level: * = P < 0.001; ** = P < 0.01; *** = P < 0.05;

- = Not applicable.

Source: Authors calculations from the Swaziland Demographic and Health Survey 2006–07.

### Antenatal care

The results show that 9 out of 10 women (97.3%) in their respective reproductive ages went for antenatal visits, particularly those aged 35–39 with the proportions of 98.8%. The utilization of antenatal services was also higher among women who have given birth to less than three children (98.2%) and lower among those with six or more children (95.1%). Hence, parity is significant with the use of antenatal services, with χ^2^ = 20.2 and P < 0.001. Furthermore, women who watch television are far more likely to utilise these services (χ^2^ = 30.1 and P < 0.001) followed by those who are exposed to the radio (χ^2^ = 27.8 and P < 0.001). Again, factors such as maternal education and wealth quintile were associated with the use of antenatal services. The evidence is provided by their lower p-values (P < 0.001). The rate of antenatal care utilization is high among women with secondary education (98.6%), followed by those with higher education (97.2%). Considering the wealth quintile, women from the rich quintile (98.8%) tend to use antenatal services more compared to those from the poor quintile (95.6%). The multivariate analysis shows that women aged 30–34 are more likely to use antenatal care [OR = 1.63, 95% (C.I: 0.1 - 27.9)] compared to women aged 15–19. Women aged 44–49, have a lower odds ratio [OR = 0.44, 95% (C.I: 0.0 - 15.1)], which means they are less likely to use antenatal services compared to women aged 15–19 years. In relation to parity, women who have 3–5 children are about five times more likely [OR = 4.90, 95% (C.I: 0.5 - 52.7)] to use antenatal services than those with 1–2 children. The results show that media exposure is related to odds of using antenatal services. For instance, women who listen to the radio every-day are about two times more likely [OR = 1.99, 95% (C.I: 0.6 - 7.0)] to use antenatal care than those who do not listen to the radio at all. Women from the middle-wealth quintile are times more likely [OR = 1.68, 95% (C.I: 0.3 - 8.3)] to use antenatal care than those who are classified as being poor. In addition, women who are literate were more likely [OR = 1.84, 95% (C.I: (0.5 - 7.3)] to use antenatal care than those who are illiterate. Also, women who reside in urban areas were more likely to use antenatal care than those who reside in rural areas [OR = 0.66, 95% (C.I: (0.2 - 2.8)]. There is a significant relationship between use of antenatal care and being informed of pregnancy complications (P < 0.05).

### Delivery care

There is a high use rate of delivery care in the country, with 74.0% of women using institutional deliveries, as compared to the 26.0% who still use non-institutional deliveries. Regarding delivery care, all the socio-demographic factors included in the study (i.e. *woman’s age, parity, mass media, maternal education, wealth quintile, literacy,* and *residence* were statistically significant with delivery care, and have chi square (χ^2^) values ranging from 20.1 to 302.7 and P < 0.001. More than 70% of women aged 15–34 tend to give birth in institutions, though this trend declines as women grow older. Most women (82.0%) with less than three children prefer giving birth in institutions compared to those with six or more children (at 55.5%). The results also show that institutional deliveries are higher among women who watch television, read newspapers and listen to the radio almost every-day with the proportions of 90.0%, 89.2% and 77.2% respectively. With regard to maternal education, 96.0% of women with higher education prefer institutional deliveries, followed by 83.4% of those with secondary education. Likewise, regarding wealth quintile, 89.3% of women from the rich quintile deliver in institutions as compared to 57.5% of those from the poor quintile. The same trend is seen among women who are literate and those living in urban areas with 76.2% and 88.5% respectively.

Table 4 shows that media exposure also relates to the odds of using delivery care. Women who read the newspaper almost every-day are more likely to use delivery care than those who do not read at all; while those who read less than once a week are less likely to use delivery services [OR = 0.84, 95% (C.I: (0.4 - 1.7)]. Similarly, women who watch television almost every-day are almost two times more likely to use this service than those who do not watch television. Women who watch less than once a week are less likely to use delivery care [OR = 1.33, 95% (C.I: (0.5 - 3.6)]. Concerning maternal education, women with higher education are more likely to use delivery care than those with no education. Pertaining to the wealth quintile, women from the rich quintile are more than twice as likely [OR = 2.55, 95% (C.I: 1.3 - 4.9)] to use this service compared to those who are classified as being poor. Furthermore, women with middle wealth quintile are less likely to use delivery care than those in the poor wealth quintile; where women in middle-wealth and rich quintiles are associated with the use of delivery care, at P < 0.05 and P < 0.001 respectively. In addition, women who are literate are almost twice more likely [OR = 1.99, 95% (C.I: 0.7 - 5.5)] to use delivery care than those who are illiterate. Women who are resident in rural areas are less likely [OR = 0.82, 95% (C.I 0.4 - 1.5)] to use delivery care compared to their counterparts in urban areas. Women aged 45–49 are less likely [OR = 0.27, 95% (C.I: 0.0 - 2.8)] to use delivery care than those in the reference group (15–19). In terms of parity, women with six or more children are less likely [OR = 0.41, 95% (C.I: 0.2 - 1.0)] to use the service compared to those with 1–2 children.

### Postnatal care

It can be established that postnatal care is not being completely utilized in the country; this is evidenced by the low 20.5% use rate – meaning that over three-quarters of women do not use this service. The results emanating from the bivariate analysis revealed that only one out of the ten factors has a significant relationship with postnatal care. Wealth quintile has a lower p-value (P < 0.05), signifying existence of a relationship. The proportions of women receiving postnatal services are more prevalent among those aged 35–39 years (28.6%). The results further indicate that utilization of postnatal services is high among women who have had less than six children. The rate of postnatal care utilization is low among women who have had six or more children (16.8%). Considering the low use rate of postnatal services in the country, women who have used postnatal care have access to the media. The majority of women indicated that they watched television at least once a week (39.4%), and some watched almost every-day (31.0%). Regarding maternal education, 27.8% of women with secondary and 25.0% with higher education revealed that they use postnatal services. The same trend of higher rates of use is seen among those who are literate (22%) and those staying in urban areas (23%). Furthermore, 31.5% of women in the rich quintile use postnatal services more as compared to those in the middle-wealth quintile (15.0%). The multivariate analysis revealed that women aged 35–39 are almost three times more likely (C.I: 0.3 - 24.2) to use postnatal care than those aged 15–19. Having less than six children increases the odds of using postnatal care. Women with 3–5 children are 1.08 times more likely (C.I: 0.4 - 2.7) to use postnatal care than those with 1–2 children; whereas, women with six or more children are less likely to use postnatal services [OR = 0.44, (C.I: 0.1 - 1.5)]. Furthermore, maternal education increases the odds of using postnatal services, where women with secondary education are 1.58 times more likely (C.I: 0.4 - 6.6) to use postnatal care than those with no formal education. In relation to residence, women in rural areas are times more likely [OR = 1.35, 95% (C.I: 0.4 - 4.1)] to use postnatal care than those in urban areas. This might be due to the health service providers taking steps to deliver these services to the rural communities. Women who watch television at least once a week are over four times more likely [OR = 4.36, 95% (C.I: 1.5 -12.7)] to use this service than those who do not watch at all. Likewise, women who are literate are more likely [OR = 1.35, 95% (C.I: 0.4 - 5.1)] to use postnatal services that women who are illiterate.

### Immunization

Most women do immunize their children – with 88.8% of women revealing that they have immunized their children in the five years preceding the survey. It is very important for mothers to ensure that their new-born babies are immunized very often so they could grow healthier and stronger without any harmful diseases. The results revealed that four factors (literacy, P < 0.05; exposure to the radio, P < 0.05; residence, P < 0.01; and wealth quintile, P < 0.001) are correlated with immunization. In relation to the rates of immunization, eight in ten mothers of reproductive age got their children vaccinated, particularly those between ages of 35–49. The majority of women who: have 3–5 children (90.5%); have primary education (90.1%); are from the middle-socio-wealth quintile (96.0%); are literate (89.5%); reside in urban areas (93.8%) – have immunized their children. In addition, non-use of immunization is high among children whose mothers: are aged 20–24 (11.6%); have six and more children (18.1%); have no education (16.7%); and are from the poor quintile (20.0%). The regression analysis revealed that children whose mothers are aged 40–44 are more likely [OR = 17.72, 95% (1.3 - 250.1)] to use immunization than those whose mothers are aged 15–19. Women in the age groups 35–39 and 40–44, as well as those with six or more children are associated with use of childhood immunization (P < 0.05). With regard to parity, children whose mothers have 3–5 children are more likely [OR = 1.23, 95% (C.I: 0.5 - 2.8)] to use immunization than those with 1–2 children. Children whose mothers have six or more children are less likely to be immunized [OR = 0.20, 95% (0.0 - 0.9)] than those who have 1–2 children. Moreover, children whose mothers have primary education are more likely [OR = 1.41, 95% (C.I: 0.3 - 6.3)] to use childhood immunization than those whose mothers have no education. The results also show that the use of immunization decreases as the educational levels increase. In terms of the wealth quintile, children from the middle-wealth quintile are over five times more likely [OR = 5.28, 95% (C.I: 1.7 - 16.3)] to be immunized than those from the poor quintile; where middle-wealth and rich quintiles are associated with the use of childhood immunization, at P < 0.001 and P < 0.05 respectively. Children whose mothers are literate [OR = 1.83, 95% (C.I: 0.4 - 7.4)], and those whose mothers reside in urban areas are more likely to be immunized. Inversely, children whose mothers listen to the radio less than once a week are almost twice as likely [OR = 1.97, 95% (C.I: 0.5 - 7.9)] to be immunized than those whose mothers do not listen to the radio.

## Discussion

This study is based on the SDHS of 2006–07, which was the first of its kind in the country in terms of coverage. It is an explorative and descriptive study, which aimed to examine the factors that influence maternal and child healthcare utilization in the country. Generally, the results revealed that the country has a high use rate of maternal (except for postnatal care) and child healthcare services. Table 1 shows that there is a high use rate of antenatal and delivery care and a low rate of postnatal care use. Immunization uptake is also high, averaging more than 80%.

### Factors influencing the use of maternal healthcare services

The study found that woman’s age has a significant influence on antenatal and delivery services. According to the bivariate analysis, women aged forty and above use maternal healthcare services less than those younger than forty. The bivariate analysis revealed that the use of institutional deliveries decreases with age, whereas use of non-institutional deliveries increases with woman’s age. Mostly young women use institutional deliveries compared to adults. For instance, the majority of women aged 15–19 deliver in institutions while the rate is lower for those aged 45–49. Furthermore, use of maternal health services is high among women with fewer children; and there is a significant relationship between parity and the use of maternal healthcare services (particularly antenatal care and delivery care). For instance, there is a high utilization rate of maternal health services among women with less than six children. The analysis revealed that use of institutional deliveries decreases with parity – where women with fewer children use delivery care more than women with six or more children. Literature shows that older women (and those with higher parity) tend to use maternal health services less frequently than younger women with fewer children [7,11]; older women who have given birth before tend to believe that they are better equipped to handle their pregnancy [25,26] – this could explain the minimal use of maternal health services among those aged forty and above and those with more than six children.

Access to information about maternal health services can considerably increase the rates with which these services are being used. The study findings illustrate a clear indication that exposure to mass media (i.e. newspapers, radio and television) plays a major role in informing women about maternal health services. Mass media is associated with use of antenatal and delivery care. For instance, women who listen to the radio every-day had higher odds of using antenatal care than those who listen less than once a week. Moreover, use of institutional deliveries increases with media exposure – where women who are not exposed to the media use maternal health services less frequently than those exposed daily. This is also revealed by the regression model findings, where it becomes clear that women who are exposed to the media on daily basis are more likely to use delivery care than those not exposed to the media. Various studies have argued that exposure to mass media influences the use of maternal health services [8,25]. Apart from that, the bivariate findings indicate that being informed about pregnancy-related complications is associated with delivery and antenatal care.

In addition, the findings also show a significant relationship between maternal education and the use of antenatal and delivery care. There is a high rate of maternal healthcare utilization among women with secondary and higher education, where nine in ten women use antenatal care. Moreover, the findings revealed that the use of institutional deliveries increases with maternal education, whereby women with higher education use delivery care more than those with no education. The influence of maternal education on the use of maternal healthcare services is further illustrated by the regression model, where women with secondary education are more likely to use postnatal care than those with no formal education. With regard to literacy, literate women use institutional deliveries more than those who are illiterate. Women’s literacy levels are associated with the use of antenatal and delivery care. Studies have supported this finding, and have argued that maternal education and women’s literacy significantly influences the use of maternal healthcare [11,26,27].

According to the study findings, the wealth quintile is statistically associated with the use of maternal healthcare services. Likewise, the use of maternal healthcare also increases with the wealth quintile of women – where women from the rich quintile use maternal health services more than those from the poor quintile. Non-use of maternal health services is thus very high in the poor quintile. Middle-wealth and rich quintiles are associated with the use of delivery care. Moreover, women from the rich quintile are more likely to use delivery and postnatal care. Likewise, women from the middle-wealth quintile are more likely to use antenatal care. These findings are supported by various studies which have found that women’s wealth (or socio-economic) status influences the use of maternal health services [7,8,12]. Furthermore, the findings show that type of place of residence is associated with delivery care. The bivariate analysis revealed that women in urban areas use maternal health services more than those in rural areas, even though most of the women in the study sample are from rural areas. This finding is supported by various authors who have noted that there are differentials in maternal healthcare use between rural and urban areas, where women in urban areas use maternal health services more than those in rural areas [12,19]. Inversely, the regression model revealed women in rural areas are more likely to use postnatal care than those in urban areas.

### Determinants of use of childhood immunization

There are high rates of childhood immunization among women aged 35–49 and those with less than six children. The bivariate and multivariate analyses revealed that women aged 40–44 are more likely to immunize their children than younger women. These findings are supported by a study which found that 6 in 10 children whose mothers were aged 45–49 were fully immunized in Uganda, compared to only 3 in 10 children whose mothers were in the 15–19 age-cohort [15]. Moreover, women in the age groups 35–39 and 40–44, as well as those with six or more children are associated with use of childhood immunization. Women aged 35–44 are significantly correlated with the use of immunization. There was an inverse relationship between use of immunization and maternal education whereby the higher the education of women, the lower the immunization of children. Women with primary education have higher rates of using immunization. This finding is validated by the regression model, where children whose mothers have primary education are more likely to be immunized than those whose mothers have higher education. This is different to other studies which have found that use of immunization is higher among children whose mothers have secondary or higher education [16,17]. This difference might be caused by country-specific behaviour patterns related to use childhood immunization. Regarding mass media, listening to the radio is associated with childhood immunization. The findings indicate that reading the newspaper increases the chances of using childhood immunization. Wealth quintile is also associated with immunization. The bivariate and multivariate analyses reveal that utilization rates of immunization are high among women from the middle-wealth quintile. Moreover, women from middle-wealth and rich quintiles are associated with the use of childhood immunization. Literacy is also significantly correlated with immunization. The study findings indicate that literate women have higher rates of immunizing their children than those who are illiterate. Furthermore, place of residence has a significant association with childhood immunization, where women in urban areas have higher use rates than those in rural areas.

## Conclusion

Even though there is a high utilization rate of maternal and child healthcare services in Swaziland – the use of postnatal services is minimal. The low use of postnatal services could be explained by that women tend to believe that it is not necessary to go back for check-ups after delivery, unless complications arise. It is at this level that most maternal deaths could occur; therefore postnatal care is an important dimension of maternal (and child) health care that should not be overlooked. Hence, we suggest that programs to educate families about the importance of maternal (particularly postnatal care) and child healthcare services should be implemented. This will then give women (and their families) more knowledge about the importance of these services. Even though the utilization of antenatal, delivery and immunization services is quite high, there is room for improvement. Initiatives relating to maternal and child health tend to focus only on women, but we believe that the family, as a cohesive unit, should be the main focus for interventions. Pregnancies are supposed to be inclusive where both men and women participate to ensure a healthy mother and child. Interventions should focus on the points mentioned above, and more particularly on: (a) age differentials in use of maternal and child health services, (b) women with higher parities, (c) women in rural areas, and (d) women from the poor quintile. We also suggest that future studies could use the qualitative approach to study issues associated with the low use of postnatal services.
